# Automated Digital Image Optimisation in Intraoperative 2D and 3D Imaging Using a Mobile C‐Arm With Flat‐Panel Detector

**DOI:** 10.1002/rcs.70053

**Published:** 2025-02-27

**Authors:** J. Groh, M. Perl, L. Bräuer, H. Stadthalter

**Affiliations:** ^1^ Department of Trauma and Orthopaedic Surgery Friedrich‐Alexander‐Universität Erlangen‐Nürnberg Universitätsklinikum Erlangen Erlangen Germany; ^2^ Institute for Functional and Clinical Anatomy Friedrich‐Alexander‐Universität Erlangen‐Nürnberg Erlangen Germany

**Keywords:** automated image analysis, intraoperative imaging, MAR, metal artifact reduction, ScrewScout, SpotAdapt

## Abstract

**Background:**

Assistance tools for intraoperative 2D and 3D imaging to decrease acquisition effort and to improve assessment of 3D image data were evaluated.

**Methods:**

Two automated optimisation procedures were evaluated in a cadaver (Cios Spin, Siemens, Germany): The ScrewScout function for assisted pedicle screw assessment. Then, an algorithm for metal artefact reduction (MAR).

Additionally, a tool for simplified setting of image contrast and brightness was evaluated regarding the result and elapsed time.

**Results:**

The time required without automated assistance was 83s [70–105]. With the computer assistance, this time was significantly lower at 22s [15–32] (*p* = 0.003). MAR resulted in an improvement in image impression. This improvement became smaller with increasing clinical experience. The time needed for setting of image acquisition parameters was significantly (*p* = 0.05) lowered from 140s [24–389] to 61s [14–166] using the assistance tool.

**Conclusions:**

Automated assistance tools for image optimisation can provide practical support in the intraoperative setting.

## 1 Introduction

Intraoperative imaging is an essential part of orthopaedic and trauma surgery, and intraoperative 2D and 3D imaging is playing an even more decisive role with the increasing use of minimally invasive surgical techniques [1]. In particular, 2D and 3D imaging plays an essential role in minimally invasive percutaneous spinal surgery [2].

Mobile C‐arm systems equipped with flat‐panel detectors (FPD) are commonly used in orthopaedic, trauma, and neurosurgery procedures. These systems can provide high‐quality images of the patient's anatomy, which can be used to guide the surgeon during the procedure—not only in spinal surgery but throughout different clinical specialities [3, 4]. However, obtaining high‐quality images using C‐arm systems can be challenging due to several factors, including the patient's anatomy, the position of the C‐arm, and the X‐ray beam angle. Furthermore, the quality of the images obtained can be influenced by factors such as radiation dose, noise, and artefacts.

Image optimisation is a crucial step in obtaining high‐quality images using mobile C‐arm systems equipped with FPD. Image optimisation involves the adjustment of various imaging parameters, such as exposure, contrast, and brightness, to achieve the desired image quality. The optimisation of these parameters can be a complex process that requires a deep understanding of the imaging system and the underlying physics of X‐ray imaging.

Major challenges regarding intraoperative imaging are the time and technical know‐how required to optimise the image quality, reduce metal artefacts and evaluate the results of the 3D scans in the OR [5].

In this study, several techniques of automated image optimisation in intraoperative 2D and 3D imaging were evaluated. The optimisation of imaging parameters is crucial for achieving high‐quality images that can guide the surgeon during the procedure. The techniques presented in this paper can help improve the accuracy and safety of surgical procedures while minimising radiation exposure to the patient and the surgical team.

## 2 Materials and Methods

### 2.1 Ethics Statement

The study was carried out on two donor bodies. The use of the body donor was carried out in collaboration with the Anatomical Institute. Consent for the use for scientific purposes was given by the individual during their lifetime in accordance with the standards of the Anatomical Institute.

According to clincal standards in the procedure of posterior percutaneous minimally invasive instrumentation, pedicle screws were inserted into both sides of the vertebrae Th11, Th12, L1, and L2.

**FIGURE 1:**
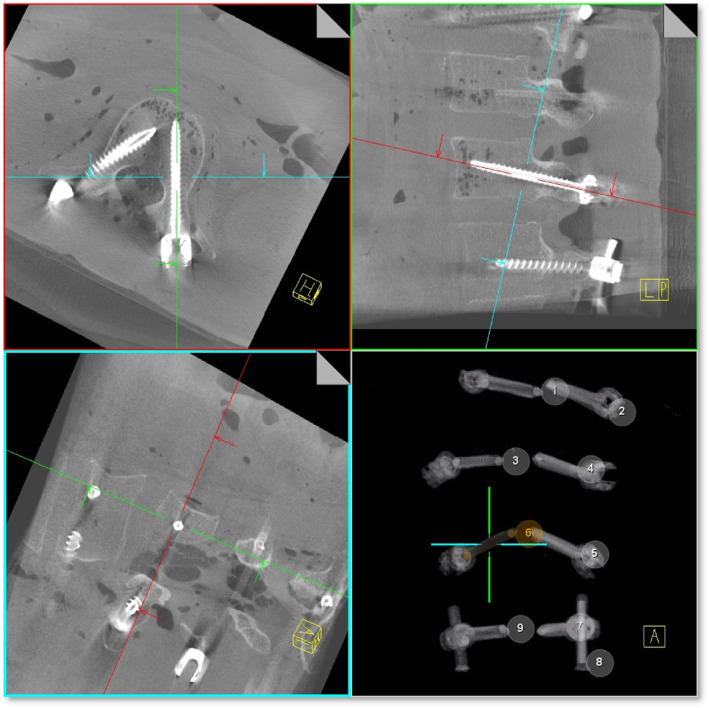
ScrewScout with MPRs automatically adjusted to pedicle screws.

**FIGURE 2:**
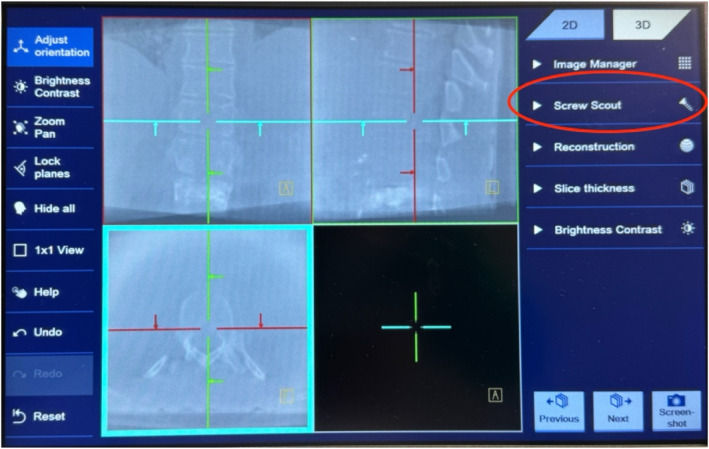
Accessing the ScrewScout application.

**FIGURE 3:**
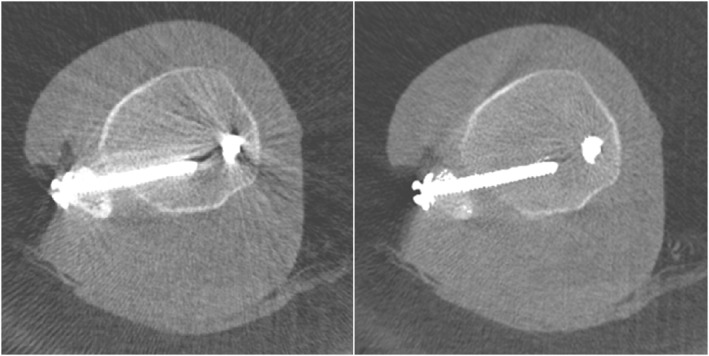
Example of a 3D scan without (left) and with MAR (right).

**FIGURE 4:**
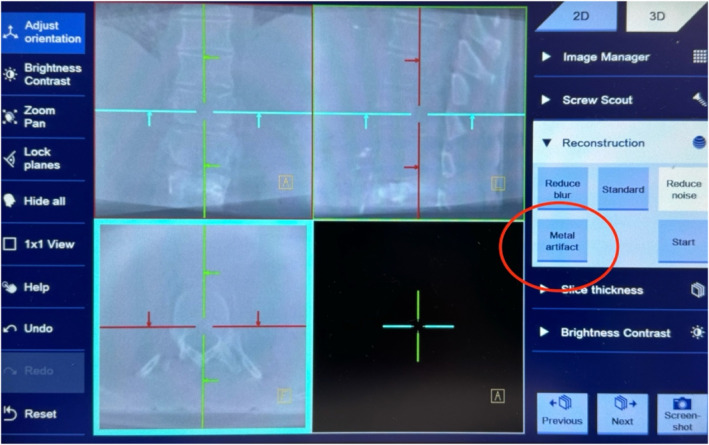
Accessing the MAR application.

**FIGURE 5:**
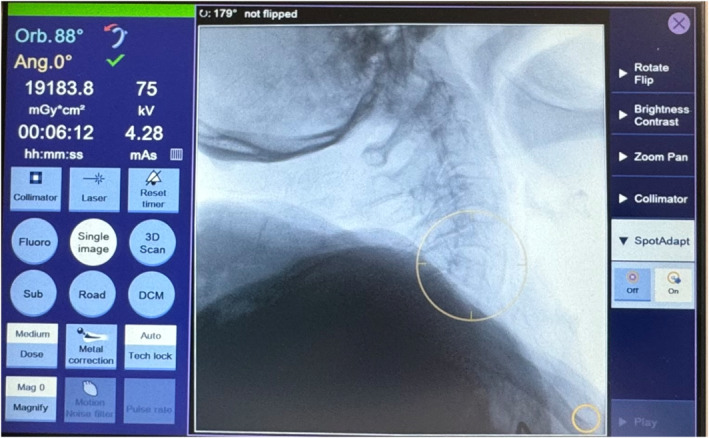
SpotAdapt in use: the yellow circle is directed to the region of interest on the touchscreen.

1. Subsequently, a mobile C‐arm (Siemens Healthineers, Cios Spin) was used to capture a 3D scan of the area of surgical intervention. Two different functions of the device (see 1.1 and 1.2) were examined by subjects with different levels of clinical experience (0–10 years of clinical experience), who were then interviewed regarding the individual functions using standardised questionnaires.
  1.1 Screw Scout: An algorithm identifies the individual screws and automatically reconstructs the image in the sagittal, axial, and coronal planes (see Figure 1). Screw Scout finds and presents the screws of the intraoperative 3D scan and spares the surgeon the manual scrolling and searching, enabling the surgeon to focus on the evaluation of the screw placements directly (see Figure 2 on how to access the application). The subjects initially performed a multiplanar reconstruction without the help of a computer and assessed the position of each of the eight screws. The same process was then carried out with the help of the Screw Scout function. In each case, an evaluation was made of the time taken to correctly reconstruct and assess all six screws. A record was also kept of the rate of false positives in the identification of screws, and the simplicity of use was assessed by the subjects (on a scale of 0–5).
  1.2 Metal artefact reduction (MAR): An algorithm detects metal artefacts and removes them from the image (for an example see Figure 3). The tool is easy to access (see Figure 4) and simplifies achieving better 3D images to help optimise the visualisation of important anatomical structures. The following parameters were each assessed with and without MAR:
^–^
overall image impression (0–5)
^–^
absence of relevant image information (0 = no, 1 = yes)
^–^
screw tip: clearly defined? (0 = no, 1 = yes)
^–^
screw edges: clearly defined? (0 = no, 1 = yes)
^–^
screw diameter (in mm)
^–^
medial pedicle wall perceptible? (0 = no, 1 = partially visible, 2 = visible over entire length of screw)
^–^
posterior wall of vertebra perceptible? (0 = no, 1 = partially visible, 2 = visible in all layers)
2. A second donor body was used to evaluate the SpotAdapt function for automatic optimisation of 2D X‐ray images. SpotAdapt enables the user to optimise the imaging quality of the POI without any radiological knowledge by simply moving a circle to the area of interest (see Figure 5). The subjects were asked to produce an X‐ray image of a defined anatomical region (cervical spine, lateral; thoracic spine, lateral; sacrum, lateral) and to manually optimise the image by altering the configuration on the C‐arm (e.g., tube voltage, pulse rate, collimation) until they considered the image to be satisfactory. The process was then carried out analogously using automatic image optimisation, and a record was made of the following parameters:
^–^
evaluation of the visibility of the point of interest (POI) of the corresponding region before and after manual or automatic image optimisation (0–5)
^–^
time required for manual or automatic image optimisation (in s)
^–^
number of images created during manual or automatic image optimisation
^–^
respective total dose for manual or automatic image optimisation (DAP in μGy·m^2^)
^–^
simplicity of use of SpotAdapt (0–5)
^–^
use in routine clinical practice (0–5)

## 3 Results

Nine participants with different clinical experiences took part in the study. Each person performed the respective tests once.

### 3.1 Screw Scout

On average, the time taken to reconstruct all eight screws and assess their position in the conventional manner was 83s [70–105] (± 14).

**FIGURE 6:**
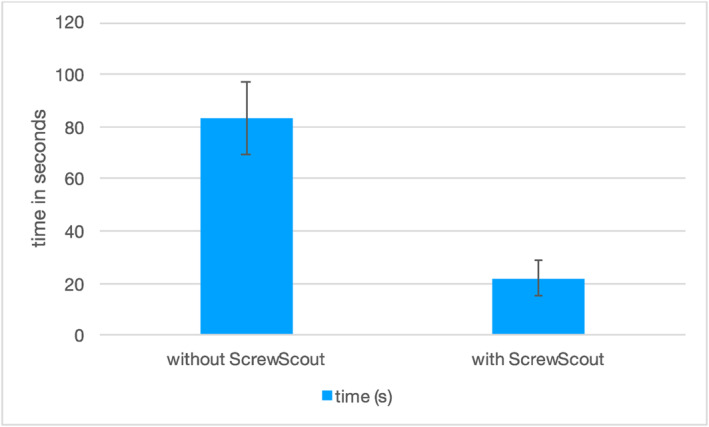
Illustrating the time difference with and without the use of the ScrewScout application.

With the Screw Scout function, the average time was 22s [15–32] (± 7). The function resulted in an average time saving of 61s [38–78] (± 15), and this figure was statistically significant (*p* = 0.003), see Figure 6. All eight screws were reliably identified in the tests.

Two false‐positive screw identifications were recorded in each test run.

No difference was observed between subjects depending on experience.

All subjects rated the simplicity of use as 5/5.

### 3.2 Metal Artefact Reduction

**FIGURE 7:**
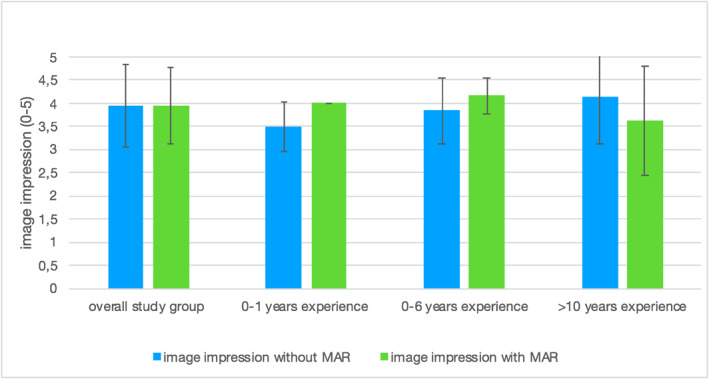
Comparison of image impression with and without MAR (rating was 0–5).

On average, the image impression was the same with and without metal artefact reduction (3.95 out of 5 in each case). However, there was a difference between the subgroups depending on clinical experience. In the group of young medical interns (0–1 years of clinical experience, *n* = 3), image impression improved by 0.5 points (3.5/5 vs. 4/5, *p* = 0.02) with MAR. In the group with 0–6 years of experience (*n* = 3), there was an improvement of 0.4 points (3.8/5 vs. 4.2/5, *p* = 0.02) in image impression. In the group with more than 10 years of experience (*n* = 3), image impression deteriorated by 0.5 points (4.1/5 vs. 3.6/5, *p* = 0.05) with MAR, see Figure 7.

**FIGURE 8:**
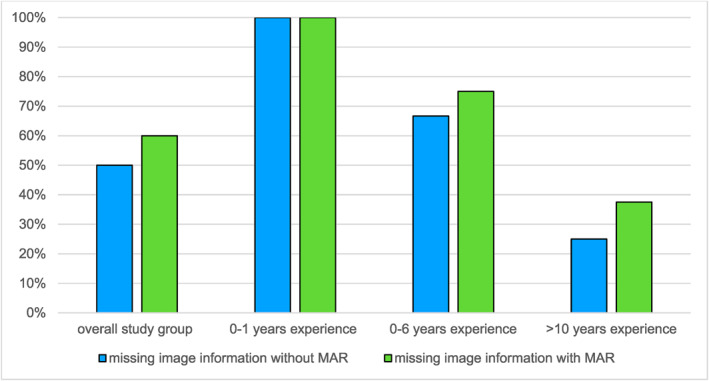
Missing image information with and without MAR.

Similar difference was observed between subgroups when it came to the absence of relevant image information (see Figure 8). In the overall study group, some relevant image information was observed to be missing with MAR. The group of young medical interns (0–1 years of clinical experience) reported a subjective absence of relevant image information both without and with MAR.

In the group with 0–6 years of clinical experience, the subjective absence of image information was reported in 67% of images without MAR and 75% with MAR (*p* = 0.17). In the subgroup with more than 10 years of experience, the absence of image information was 25% without MAR and 38% with MAR (*p* = 0.17).

Both with and without MAR, 100% of screw tips were identified.

The screw edges were visible along the entire length of the screws in 95% of cases without MAR and 100% of cases with MAR.

On average, the measured screw diameter differed by 0.3 mm (6.7 [5–8]mm (± 0.8 mm) without MAR versus 6.4 [5–8]mm (± 0.9 mm) with MAR, *p* = 0.03). The manufacturer indicated a screw thickness of 6.5 mm.

In terms of visibility of the medial pedicle, there was no improvement in image impression at the level of the overall study group (average: 1.7 with and without MAR).

On average, image impression improved from 1.4 to 1.6 (*p* = 0.09) in the subgroup of young interns (0–1 years of experience) and from 1.6 to 1.7 (*p* = 0.3) in the subgroup with up to 6 years of experience. In the subgroup with < 10 years of experience, there was an average deterioration from 1.9 to 1.6 (*p* = 0.09).

In the overall study group, the recognisability of the posterior wall improved from 0.8 without MAR to 1.1 with MAR (*p* = 0.04).

On average, the visibility of the posterior wall of the vertebral body improved from 0 to 0.5 (*p* = 0.02) in the subgroup of young medical interns (0–1 years of experience) and from 0.5 to 0.9 (*p* = 0.01) in the subgroup with up to 6 years of experience. In the subgroup with < 10 years of experience, there was no improvement in image assessability (1.3 vs. 1.3).

## 4 SpotAdapt

**FIGURE 9:**
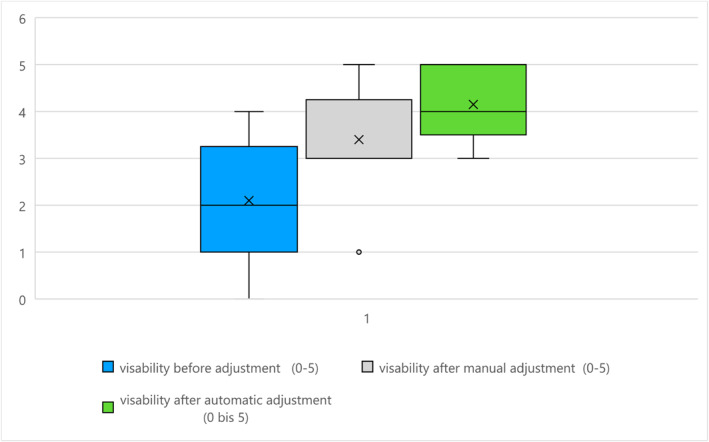
Image impression before and after image optimisation.

Before image optimisation, the average reported image impression was 2.1 [0–4] (± 1.3). After manual image optimisation, there was an improvement by 1.3–3.4 [1–5] (± 1.1), and this improvement was statistically significant (*p* = 0.02). An illustration is given in Figure 9. After automatic image optimisation, image impression improved to 4.2 [3–5] (± 0.7); this improvement was highly statistically significant (*p* < 0.001). Overall, image impression was significantly better (*p* = 0.05) following automatic optimisation with SpotAdapt.

**FIGURE 10:**
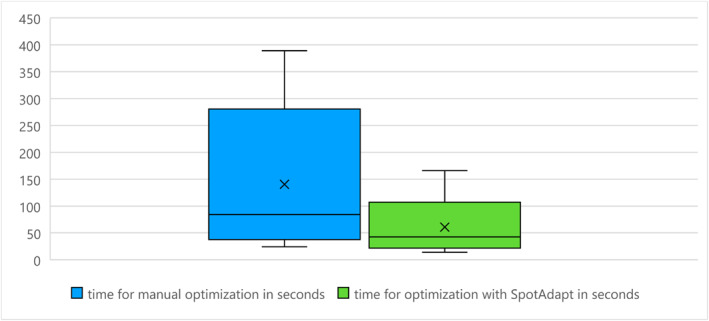
Time saving with SpotAdapt.

On average, manual configuration of the image took 140s [24–389]s (± 128s), and automated optimisation took 61s [14–166]s (± 47s). The average time saving of 79s with SpotAdapt was statistically significant (*p* = 0.05), see Figure 10.

The number of X‐ray images produced in the procedure was 1.6 less with SpotAdapt (manual: 4.8 [1–13] (± 3.7); automatic: 3.2 [1–10] (± 2.7)), but this difference was not statistically significant (*p* = 0.17).

**FIGURE 11:**
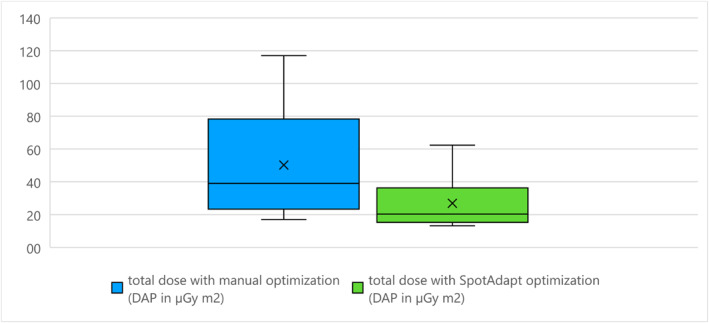
Dose reduction with SpotAdapt.

**FIGURE 12:**
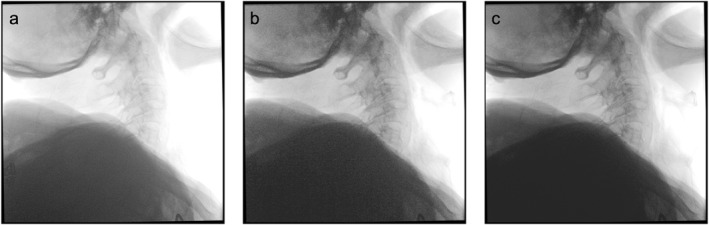
Lateral view of the C‐Spine without optimisation (a), after manual optimisation (b) and after optimisation with SpotAdapt (c).

The dose area product (DAP) was 50 μGy m^2^ [17–117] μGy·m^2^ (± 34 μGy m^2^) with manual optimisation and 27 μGy m^2^ [13–62] μGy·m^2^ (± 15 μGy m^2^) with automatic configuration, and the dose reduction due to the SpotAdapt function was statistically significant (*p* = 0.04). For the results see Figure 11, a clinical example is given in Figure 12.

All subjects rated the function's operation as good (4.3 [4–5] (± 0.5)) and approved of its use in routine clinical practice (4.3 [4–5] (± 0.5)).

## 5 Discussion

As intraoperative imaging devices have reached a very high degree in producing high‐quality images that allow for proper assessment of relevant factors in spinal surgery, one of the upcoming fields of evolution will surely be the automatic optimisation of the visualised image data [6]. Although the fully automated and highly reliable evaluation of image data is still a thing for the future, partial steps can already be done in an automated manner [7]. In this paper, two 3D‐ and one 2D‐applications were examined regarding their benefits in a realistic clinical setting.

The Screw Scout application is intended to take over routine tasks from the surgeon to ease evaluation and save precious operation time. In a preliminary study, it was already shown to achieve a recognition rate that allows for routine usage by Beisemann et al. [8]. In the current, more clinical setting, it was possible to demonstrate significant time savings in the assessment of screw positioning as well as offering a reliability comparable to that of conventional, manual reconstruction. Moreover, the function requires less prior knowledge and can therefore be used intraoperatively even by medical support staff and then demonstrated to the surgeon. Accordingly, there is no need for the surgeon to operate the C‐arm terminal or to apply a sterile cover to the terminal, which resolves sterility issues that occur when there is a need of direct interaction of the surgeon with the device.

Following from the data, it can be assumed that the patient benefits from a reduced operating time and a lower risk of contamination due to the usage of the application. The function was evaluated positively by all subjects with a wide range of experience levels.

In terms of metal artefact reduction, results vary significantly between subgroups depending on their level of experience in spinal surgery and accompanying intraoperative imaging.

While subjects with limited clinical experience reported a better image impression with artefact reduction, the opposite was found among those with many years of experience. Although the function reduces image noise and artefacts, the way of function of the system also means that it might remove regions of the image that appear to help experienced surgeons with decision‐making. The effects of other MAR techniques have been described recently in a retrospective clinical study with a fixed C‐arm setting by Yann et al. [9]. The authors describe a significant enhancement of assessability thanks to the MAR. Fixed C‐arms do have several advantages over mobile C‐arms regarding image information and quality, so the power of MAR tools is naturally higher. Regarding mobile C‐arms, there are several approaches to further increase the effects of MAR, such as modified (non‐circular) trajectories or the inclusion of implant information (known‐component) [10, 11].

In our setting, MAR resulted in better and more artefact‐free imaging of the metal itself in no differences between subgroups.

Studies, regarding the effort of time and (unnecessary) additional acquisitions to achieve image optimisation are missing. There has been a certain attention due to the introduction of flat‐panel detectors that enabled higher image quality by reducing dosage at the same time [12, 13, 14]. The use of the SpotAdapt application resulted in a significant improvement in image impression while also reducing the number of X‐ray images required to achieve an optimal acquisition. Subsequently, this led to a significant time saving and dose reduction. Radiation dose is an utmost important factor to evaluate in intraoperative imaging, as the surgical team is exposed to scatter radiation from each acquisition [15, 16, 17]. Therefore, the patient benefits from reduced operating time, increased safety, and improved radiographic visibility as well as reduced intraoperative radiation exposure.

## 6 Conclusion

Assistance tools for image optimisation can provide intraoperative support and simplify processes as well as reduce operation times. The examined systems led to reduced operating time for the patient due to time savings and improved image quality. Repositioning control and control of the implant position is improved by the increased image quality; any incorrect positioning and inadequate repositioning can thus be deleted and revision operations for the patient subsequently avoided. Especially in routine tasks—as optimisation of the acquisition parameters or in MPR adjustments to assess specific implants—automated image processing can significantly save time and effort intraoperatively. Metal artefact reduction has already shown great results in improving assessability. Further integration of physical principles and information will even more improve capabilities in intraoperative assessment of implant placement and reduction.

## Author Contributions

J.G. wrote the manuscript and conducted relevant parts of the study. M.P. assisted in conducting the measurements and proofread the manuscript. L.B. organised the study and assisted in defining the study parameters. H.S. assisted in writing the manuscript, defining study parameters and conducted the measurements.

## Ethics Statement

Consent for the use of specimens for scientific purposes was given by the individual during their lifetime in accordance with the standards of the Institute for Functional and Clinical Anatomy, Friedrich‐Alexander‐University Erlangen‐Nuremberg.

## Conflicts of Interest

The authors declare no conflicts of interest.

## Data Availability

Data are available on reasonable request.
